# An Exploratory Study Investigating the Intraoral Thermal Features in Advanced Periodontitis Using Machine Learning Techniques

**DOI:** 10.3390/healthcare14213749

**Published:** 2026-11-03

**Authors:** Ratna Bhaskar Pallipamula, Siva Padmavathi Donepudi, Kopparapu Jahanavi, A. Brindha, Kishore Kumar Katuri, Ravindranath Dhulipalla, K. A. Sunitha

**Affiliations:** 1 Department of Electronics and Communication Engineering, School of Engineering and Science, SRM University–AP, Amaravati 522240, Andhra Pradesh, India; ratnabhaskar_p@srmap.edu.in (R.B.P.); sivapadmavathi_donepudi@srmap.edu.in (S.P.D.); jahanavi_kopparapu@srmap.edu.in (K.J.); 2 BioMedical Research and Artificial Intelligence Network Laboratory, SRM University–AP, Amaravati 522240, Andhra Pradesh, India; 3 Department of Electronics and Instrumentation Engineering, SRM Institute of Science and Technology, Kattankulathur 603203, Tamil Nadu, India; brindhaa@srmist.edu.in; 4 Department of Periodontology, Sibar Institute of Dental Sciences, Takkellapadu, Guntur 522509, Andhra Pradesh, India; drkishorekaturiperio@sids.ac.in (K.K.K.); drravindranathdhulipallaperio@sids.ac.in (R.D.); 5 Department of Health Engineering, School of Engineering and Science, SRM University–AP, Amaravati 522240, Andhra Pradesh, India

**Keywords:** periodontitis, non-invasive screening, periodontal screening, thermal imaging, intraoral infrared thermography, probing pocket depth (PPD), clinical attachment loss (CAL), sulcus bleeding index (SBI), machine learning techniques

## Abstract

Background/Objective: Periodontitis is a chronic inflammatory disease requiring reliable assessment of periodontal tissue involvement. This study investigates quantitative intraoral thermal features correlating with periodontal conditions using machine learning algorithms. Materials and Methods: The study included 108 participants, comprising 54 healthy controls and 54 participants with advanced periodontitis (Stage III/IV combined). Five intraoral thermal views were obtained from each participant, yielding 540 thermal images. Quantitative features describing temperature distribution, variation, and differences between anatomical views were extracted. Eleven machine-learning classifiers were evaluated using participant-level nested cross-validation, with hyperparameter tuning performed in the inner folds and performance assessed in the outer folds. Results: Higher thermal measurements were observed across all five intraoral views in the periodontitis group, accompanied by differences in PPD, CAL, and SBI. Among the evaluated classifiers, AdaBoost showed the highest internal validation performance, with an ROC-AUC of 0.998 (95% CI: 0.992–1.00), sensitivity of 98.15%, specificity of 98.15%, and accuracy of 98.15%. The extracted thermal features provided useful discriminatory information between the two groups. Conclusion: Quantitative intraoral thermal features showed potential for the exploratory screening of advanced periodontitis by distinguishing affected individuals from periodontally healthy controls.

## 1. Introduction

Periodontal diseases are prevalent inflammatory conditions that can progressively damage the tissues supporting the teeth. An estimated 1.067 billion people worldwide are affected by severe periodontitis, with a global age-standardized prevalence of 12.50%; projections suggest that the number of cases could exceed 1.5 billion by 2050 [1]. Conventional periodontal assessment relies on clinical measures such as probing pocket depth (PPD), clinical attachment loss (CAL), bleeding assessment, and radiographic evaluation of alveolar bone loss that are minimally invasive in nature. The clinical procedures described above may cause gingival bleeding, which can be uncomfortable for participants [2,3]. Furthermore, radiographic evaluation of alveolar bone loss has a risk of harm due to radiation when it is performed multiple times [4]. These limitations highlight the need for non-invasive approaches that can screen periodontal disease. This motivates the investigation of associations between thermal features and periodontal conditions.

Periodontal inflammation develops when dental plaque bacteria activate the body’s immune response, triggering the release of inflammatory mediators that increase local blood flow and recruit immune cells into the periodontal tissues [5]. This periodontal inflammatory response can increase blood flow and lead to increased gingival surface temperature at the affected site [6]. Accordingly, greater periodontal inflammation and deeper periodontal pockets may be associated with higher subgingival temperatures. The current literature studies state that subgingival temperature is associated with clinical indicators of periodontal inflammation and tissue destruction, such that sites with greater pocket depth, bleeding on probing, plaque accumulation, gingival redness, and attachment loss tend to exhibit higher temperatures [7].

Infrared thermography has been explored across various biomedical fields, including dentistry, as a non-invasive approach for assessing, screening, and monitoring temperature variations associated with inflammation and vascular changes in conditions such as musculoskeletal disorders, vascular diseases, and other inflammatory conditions [8,9,10,11,12,13]. As summarized in Table 1, the existing literature has examined its application in gingival inflammation, periodontal tissue assessment, and other dental conditions. However, the use of quantitative thermal features derived from multiple consistent intraoral views to distinguish periodontally healthy participants from those with advanced periodontitis remains insufficiently explored. Intraoral thermal imaging provides a non-contact approach for capturing spatial temperature patterns at the gingival surface. Because periodontal inflammation is associated with biological changes within the affected tissues, alterations in the local patterns may provide complementary information about inflammation activity [14,15]. The non-contact nature of thermal imaging makes it a useful complementary approach for investigating periodontal tissue changes without replacing established clinical assessment.

Existing studies have investigated temperature differences between healthy and inflamed periodontal sites [16,17], while artificial-intelligence-assisted thermal imaging has also been explored for classifying gingival inflammation [18]. The present study addresses this gap by evaluating thermal features from five consistent intraoral views for participant-level classification of periodontal health and advanced periodontitis.

This pilot study focused on participants with clinically diagnosed advanced periodontitis and healthy controls to investigate associations between thermal features and clinical periodontal parameters. The study used machine-learning classifiers to distinguish participants with advanced periodontitis from healthy controls.

**Table 1:** Comparison of Previous Studies on Gingival and Periodontal Thermography and Machine Learning.

Study	Sample/Unit	Groups/Target	Imaging Approach	Analysis/ML	Main Finding	Key Limitation
Aboushady et al. [7], 2021	80 subjects	Apical Periodontitis group, Acute Periapical abscess group, Chronic Periapical Abscess group	Thermal Imaging	Group comparison and Kappa	Significant thermal differences among inflammatory conditions	Periapical rather than periodontal disease
Elboray et al. [16], 2026	70 subjects	Healthy group, Inflammation without CAL, and Inflammation with CAL	Thermal Imaging	ROC analysis	ROC-AUC 0.94 and 0.86 for inflammation and CAL, respectively	Site-level rather than participant-level diagnosis
Çankaya et al. [18], 2026	40 subjects	Gingival Index group, BOP group	Thermal Imaging	XGBoost with five-fold CV	Reported accuracy of 92.74%	Small, specific population; gingival inflammation rather than Stage III/IV periodontitis
Morales-Cervantes et al. [19], 2026	91 Subjects	Healthy group, Gingivitis group, and Periodontitis group	Thermal Imaging	Two-phase ML	Combined accuracy of 94.51%; thermal-only accuracy of 75.82%	Point-based approach; combined clinical variables
**Present study**	108 Subjects	Healthy group, advanced periodontitis group	Thermal Imaging	Quantitative ROI features and multiple ML classifiers	Internal classification performance evaluated using nested cross-validation	Single-centre cohort

*Abbreviations:* ROC-AUC, receiver operating characteristic-area under the curve; CAL, clinical attachment level; CV, cross-validation; ML, machine learning; PPD, probing pocket depth; ROI, region of interest.

## 2. Materials and Methods

### 2.1. Study Design and Participants

The study included 108 participants, 54 healthy controls and 54 participants with advanced periodontitis, based on their clinical periodontal assessment and the 2017 World Workshop classification framework, as summarized in Table 2. A full-mouth periodontal examination was performed, with PPD and CAL recorded at six sites per tooth. The SBI was also assessed to characterize gingival bleeding. These clinical parameters were assessed for all participants, and the measurements were aggregated across the examined teeth to obtain participant-level values for statistical analysis. Intra-examiner assessment was performed for PPD, CAL, and SBI. Participants with advanced periodontitis had a PPD of ≥6 mm and a CAL of ≥5 mm, whereas healthy controls had PPD and CAL values of ≤3 mm. The SBI ranged from 2 to 5 in the advanced-periodontitis group and 0 to 1 in healthy controls.

Five intraoral thermal views were acquired from each participant, comprising frontal, right lateral, left lateral, maxillary occlusal, and mandibular occlusal views. Thus, the dataset contained 540 thermal images (108 participants × 5 views), with five images nested within each participant. Each of the three repeated thermal images was acquired under the supervision of a qualified dental practitioner. The practitioner reviewed the visible anatomical regions, including the labial and buccal gingiva, gingival margins, interdental papillae, and gingival tissues along the maxillary and mandibular dental arches. The anterior view covered the gingiva surrounding the incisors and canines, while the lateral views included the visible gingiva around the canines, premolars, and molars. The occlusal views captured the gingival regions of the upper and lower dental arches. The dental practitioner reviewed the visible gingival anatomy in each view to identify the relevant region for thermal image assessment. For the analysis, one acquisition per view was retained for thermal feature extraction. The selection process involved assessing image sharpness, the visibility of the relevant gingival anatomy, the clarity of the Region of Interest (ROI) and the absence of blurring, unwanted reflections, or other visible artifacts. The thermal images and their corresponding color palettes were examined to identify the gingival regions of interest and distinguish them from surrounding non-gingival tissues. The dental practitioner applied these criteria consistently across all participants. With 54 participants in each group, a two-sided significance level of 0.05 and a target power of 80% provide sufficient sensitivity to detect a moderate standardized between-group effect of approximately Cohen’s d=0.55.

This investigation was carried out in accordance with institutional and national ethical norms. The Institutional Ethics Committee of Sibar Institute of Dental Sciences, Guntur, Andhra Pradesh, granted ethical approval under protocol number Pr. 683/IEC/SIBAR/2025. All participants had given written informed consent prior to the clinical examination and intraoral thermal imaging.

*Inclusion criteria.* To be included in the study, participants had to be aged 35–70 years and possess at least 20 natural teeth. The periodontitis group comprised participants with a clinically established diagnosis of advanced periodontitis, based on the 2017 World Workshop classification framework [22]. The clinical parameters considered for advanced periodontitis included PPD, CAL, and SBI [23] as shown in Table 2.

*Exclusion criteria.* Individuals were excluded if they had systemic conditions that might affect periodontal inflammation or thermal responses, including diabetes, hypertension, autoimmune disease, fever, thyroid disease, or malignancy under active treatment. Participants were also excluded if they had received periodontal treatment within the previous six months; had systemic, oral, or respiratory infection within the previous two weeks; had received antibiotics or anti-inflammatory agents within the previous four weeks; were pregnant or lactating; used tobacco or alcohol; or had fixed prostheses or orthodontic appliances that could interfere with periodontal or thermal-image evaluation.

**Figure 1:**
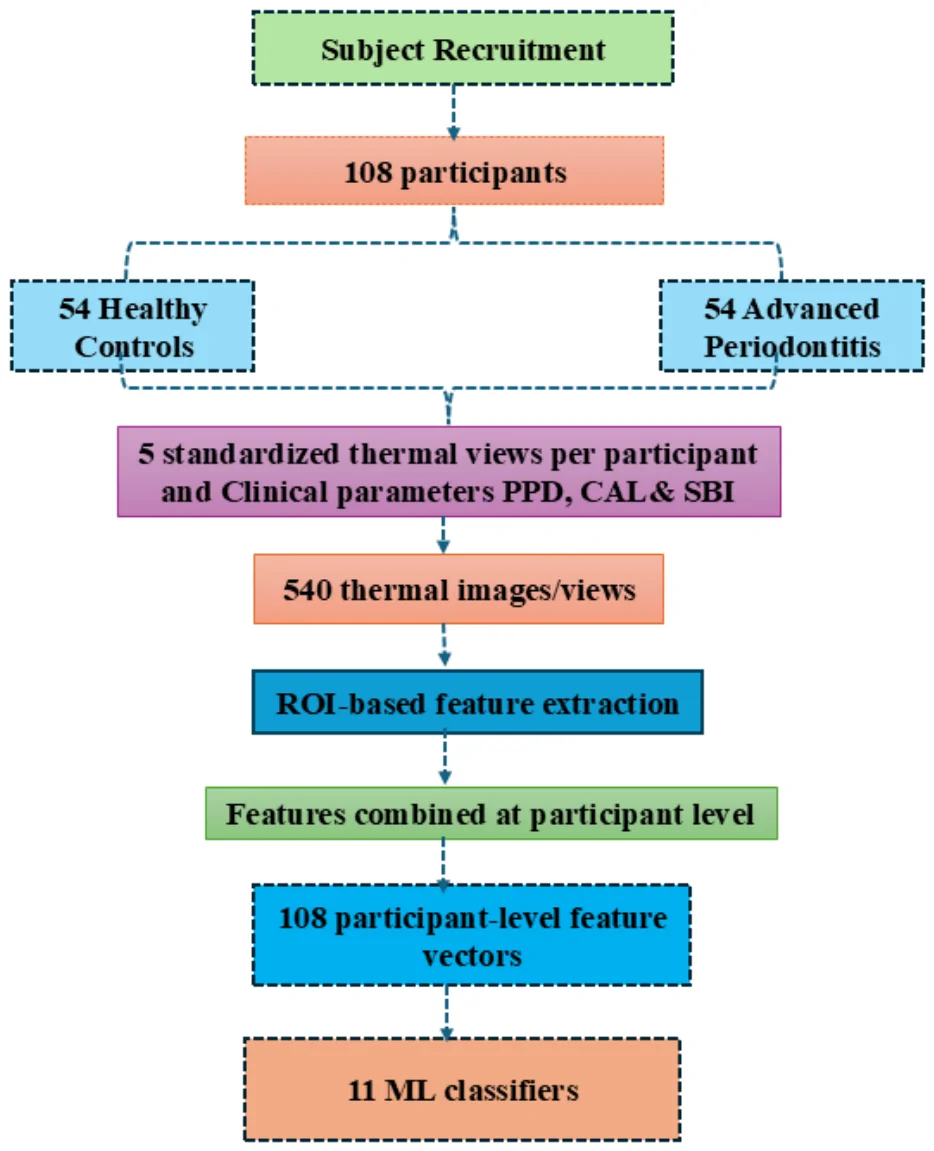
Workflow for intraoral thermal imaging and machine-learning-based exploratory classification of advanced periodontitis and healthy controls.

As shown in Figure 1, the features obtained from the five views were combined at the participant level to construct a single feature vector for each individual. Consequently, the dataset contained 108 participant-level feature vectors rather than 540 independent observations. These feature vectors were then provided as inputs to eleven machine learning classifiers to evaluate their ability to discriminate between healthy controls and participants with advanced periodontitis. This workflow distinguishes the number of participants from the number of acquired thermal images and illustrates how information from multiple anatomical views was integrated into the classification.

### 2.2. Evaluation of Clinical Periodontal Parameters

Each subject underwent a full-mouth periodontal examination by skilled periodontists, who recorded standardized clinical indicators to ensure measurement consistency. A calibrated UNC-15 periodontal probe was used to evaluate PPD and CAL at six different sites per tooth. Each participant’s PPD scores were taken from the gingival margin to the base of the pocket, while the CAL was measured from the cemento-enamel junction to the base of the periodontal pocket. Mean PPD and CAL were calculated to summarize periodontal status at the participant level. The gingival inflammatory status was evaluated using the SBI. For descriptive analysis, the mean PPD and CAL values were calculated from the full-mouth periodontal examination for each participant. These participant-level mean values were used to summarize periodontal status and were distinguished from the site-specific findings and additional clinical criteria used for periodontal staging.

The clinical periodontal assessments, including PPD, CAL, and SBI, were assessed by the clinical examiners. The SBI was assessed by gently probing the gingival sulcus and recording the presence and severity of bleeding according to the predefined SBI scoring criteria [24]. Based on these clinical findings and the 2017 periodontal classification, participants were categorized as healthy controls or patients with advanced periodontitis. Periodontitis staging considered PPD, CAL, radiographic bone loss, and disease complexity, where these assessments were available.

### 2.3. Intraoral Thermal Image Acquisition

**Figure 2:**
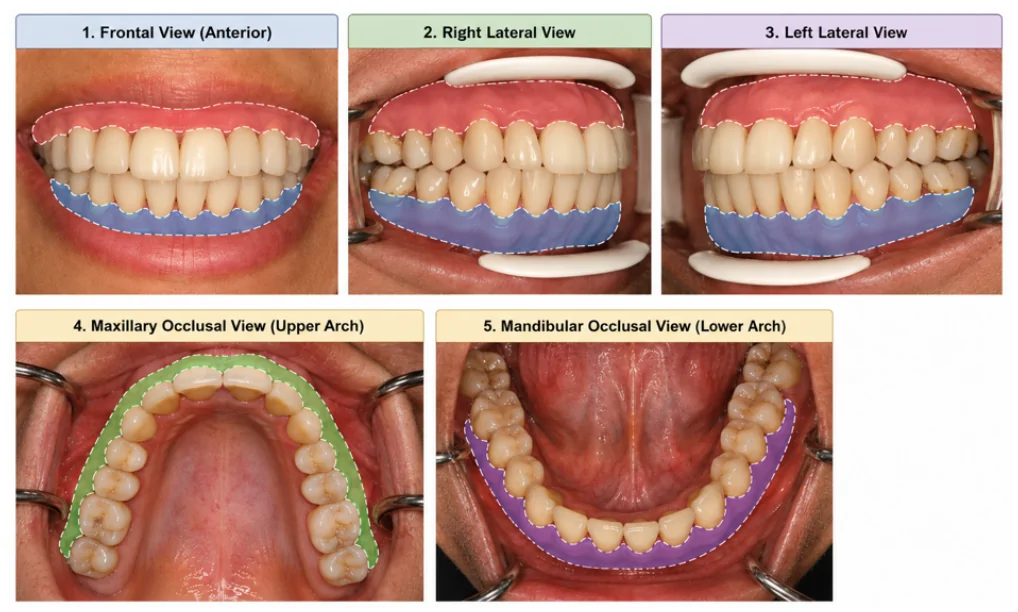
Standardized Intraoral Views and Corresponding Regions of Interest (ROIs) for Periodontal Assessment: (**1**) Frontal, (**2**) Right Lateral, (**3**) Left Lateral, (**4**) Maxillary Occlusal, (**5**) Mandibular Occlusal views.

A clinical-grade infrared camera, which can identify even slight variations in mucosal temperature brought on by periodontal infection, was used to take thermal images. To reduce the effects of environmental warmth, all imaging procedures were carried out in a temperature-controlled room at 23–25 °C [25]. Thermal image acquisition was conducted in a controlled environment, and participants were instructed to maintain normal breathing during image acquisition to reduce avoidable variation as shown in Figure 2.

**Figure 3:**
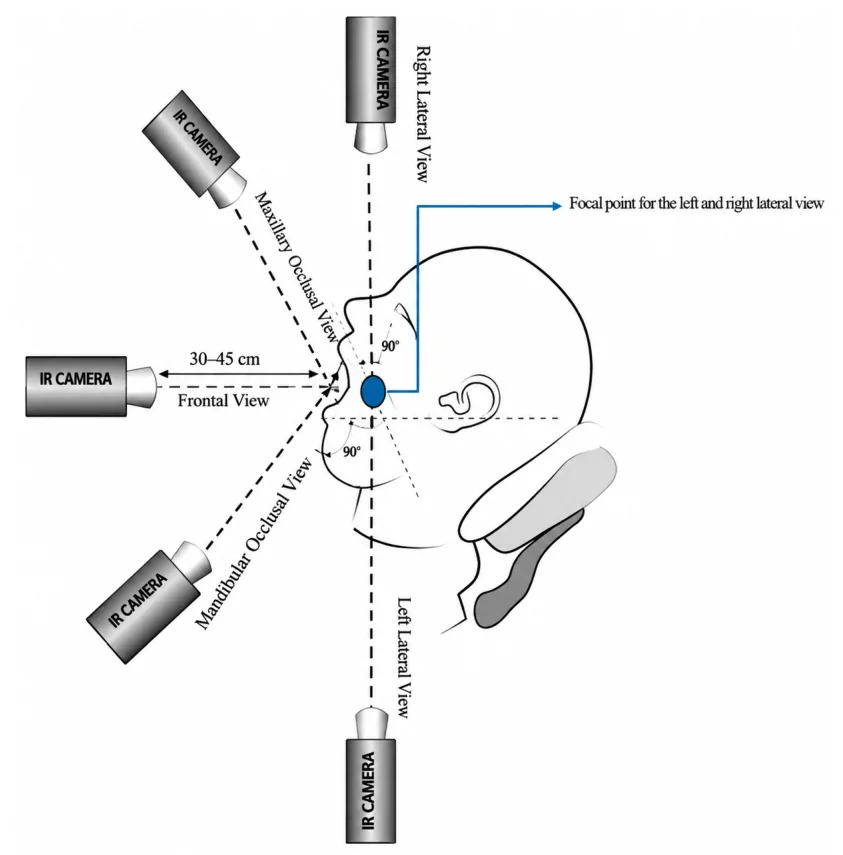
Experimental setup for intraoral thermal image acquisition using infrared thermography.

Each participant was allowed to acclimatize for 10–15 min before thermal image acquisition [17,25]. The intervals since the last food or beverage intake, oral-hygiene procedures, and recent physical activity were not quantitatively recorded during data collection and were therefore not included as controlled covariates in the present analysis. Before imaging, the dental practitioner conducted a preliminary visual examination of the teeth and gingival tissues to assess the periodontal condition and determine the participant’s clinical group. Each participant was told to slightly retract their lips to reveal gingival tissues without causing localized warmth, while the standardized camera positioning was used during image acquisition. We have also clarified that the camera was positioned within a standardized working distance in the range of 30–45 cm [26] as shown in Figure 3.

The FLIR E6 Pro thermal camera was employed in intraoral imaging because it detects subtle temperature variations related to periodontal inflammation. The device has a resolution of 240 × 180 pixels with 0.05 °C thermal sensitivity for reliably capturing gingival heat patterns, enabling the capture of spatial temperature patterns across the intraoral regions. Thermal image acquisition was performed under standardized environmental conditions. Participants were allowed to acclimatize to the room environment before imaging, and an emissivity setting of 0.98 was used throughout acquisition. These measures were intended to improve consistency across participants and reduce avoidable variation in the recorded thermal measurements. These procedures were informed by previously published studies and recommendations on standardized thermal imaging and measurement practices [27,28,29].

The acquired intraoral thermal images were analyzed using FLIR Tools software to obtain temperature measurements from the standardized intraoral views. The ROI selection were manually delineated by the dental practitioner based on the visible gingival anatomy across five intraoral views: frontal, right lateral, left lateral, maxillary occlusal, and mandibular occlusal. Because periodontal inflammation may affect different anatomical locations within the oral cavity, ROI selection involved examining the corresponding normal images alongside the thermal images and their associated color palettes. The visible gingival regions were assessed to distinguish healthy-appearing tissues from regions exhibiting signs of inflammation, thereby guiding ROI delineation. Identifiable non-gingival areas were excluded wherever possible. The dental practitioner applied this approach consistently across all participants.

In advanced periodontitis, a comparison across five oral views illustrates differences in thermal patterns compared with healthy controls, as shown in Table 3. The frontal and lateral views depict gingival inflammation, recession, and periodontal pocketing when compared to the physiologic state of health, with no such inflammation but rather normal anatomic contour and attachment. Maxillary and mandibular occlusal views depict alveolar bone loss, tooth mobility, and altered occlusion in severe cases when compared to the healthy, well-aligned dentition and intact bone in controls [30]. The regions identified as showing signs of inflammation were manually marked and circled on the thermal images for visualization and subsequent analysis. Temperatures were higher in the advanced-periodontitis group than in the healthy-control group. Together, the five views illustrate the observed thermal differences between the groups.

### 2.4. Thermal Region Selection and Feature Extraction

Intraoral thermal images were examined in five views. The regions corresponding to periodontal abnormalities were manually marked, and the resulting regional temperature values were used to construct a participant-level feature set for subsequent machine-learning analysis. The regional temperature variables were denoted by $T_{F}$, $T_{RL}$, $T_{LL}$, $T_{MO}$, and $T_{MD}$, representing the frontal, right lateral, left lateral, maxillary, and mandibular views, respectively.

The thermal features included summary statistics, regional temperature differences and ratios, standardized regional temperatures, and an asymmetry index. The summary statistics characterized the overall distribution of the five regional temperature values for each participant Equations (1)–(13). The regional comparisons were used to describe temperature variation between selected oral views.

The mean temperature represents the average thermal intensity within the selected region:

(1) $$T_{\mathrm{mean}}=\frac{1}{5}\sum_{j=1}^{5}T_{j}\;$$

where $T_{j}$ denotes the temperature of the $j^{\mathrm{th}}$ pixel.

This feature captures the range of temperature variation within the ROI:

(2) $$T_{\mathrm{range}}=\max\left(T_{1},\ldots ,T_{5}\right)-\min\left(T_{1},\ldots ,T_{5}\right)\;$$

where $T_{\max}$ and $T_{\min}$ represent the maximum and minimum temperatures within the ROI, respectively.

Thermal asymmetry measures the temperature difference between anatomically corresponding regions:

(3) $$T_{\mathrm{asym}}=\left|T_{LL}-T_{RL}\right|$$

The standard deviation (SD) quantifies the variability of temperature values within the selected ROI. It was calculated as

(4) $$T_{\mathrm{SD}}=\sqrt{\frac{1}{4}\sum_{j=1}^{5}{\left(T_{j}-T_{\mathrm{mean}}\right)}^{2}}\;$$

where $T_{i}$ represents the temperature of the *i*-th pixel within the ROI, $T_{\mathrm{mean}}$ denotes the mean temperature, and *N* is the total number of pixels included in the ROI. A higher SD indicates greater temperature variability within the selected region.

The skewness and excess kurtosis of the extracted temperature values were calculated to characterize the asymmetry and peakedness of their distributions, respectively. Skewness was computed using the third standardized moment, while excess kurtosis was calculated using the fourth standardized moment minus 3. The calculations were implemented in Python 3.12 using the statistical functions from the SciPy library.

(5) $$\gamma_{1}=\frac{\frac{1}{5}\sum_{j=1}^{5}{\left(T_{j}-T_{\mathrm{mean}}\right)}^{3}}{{\left[\frac{1}{5}\sum_{j=1}^{5}{\left(T_{j}-T_{\mathrm{mean}}\right)}^{2}\right]}^{3/2}}$$

(6) $$\gamma_{2}=\frac{\frac{1}{5}\sum_{j=1}^{5}{\left(T_{j}-T_{\mathrm{mean}}\right)}^{4}}{{\left[\frac{1}{5}\sum_{j=1}^{5}{\left(T_{j}-T_{\mathrm{mean}}\right)}^{2}\right]}^{2}}-3$$

Regional temperature differences were calculated using the following expressions:

(7) $$\begin{array}{cc} \Delta T_{F,LL} & =T_{F}-T_{LL},\\ \Delta T_{F,RL} & =T_{F}-T_{RL},\\ \Delta T_{F,MO} & =T_{F}-T_{MO},\\ \Delta T_{F,MD} & =T_{F}-T_{MD},\\ \Delta T_{RL,LL} & =T_{RL}-T_{LL},\\ \Delta T_{MO,MD} & =T_{MO}-T_{MD}. \end{array}\;$$

These differences represented frontal-to-lateral, frontal-to-arch, bilateral lateral, and maxillary-to-mandibular temperature contrasts. The corresponding regional temperature ratios were calculated as

(8) $$\begin{array}{cccc} R_{F,MO} & =\frac{T_{F}}{T_{MO}}, & R_{F,MD} & =\frac{T_{F}}{T_{MD}},\\ R_{RL,LL} & =\frac{T_{RL}}{T_{LL}}, & R_{MO,MD} & =\frac{T_{MO}}{T_{MD}}. \end{array}\;$$

Three additional temperature gradients were derived to describe differences between selected anatomical regions. The anterior gradient represented the frontal temperature relative to the mean of the maxillary and mandibular occlusal temperatures:

(9) $$G_{\mathrm{anterior}}=T_{F}-\frac{T_{MO}+T_{MD}}{2}.\;$$

The bilateral gradient represented the difference between the right and left lateral temperatures:

(10) $$G_{\mathrm{bilateral}}=T_{RL}-T_{LL}.$$

The occlusal gradient represented the difference between the maxillary and mandibular occlusal temperatures:

(11) $$G_{\mathrm{occlusal}}=T_{MO}-T_{MD}.$$

To express each regional temperature relative to the distribution of the five regional temperatures within the same participant, standardized regional values were calculated as

(12) $$Z_{j}=\frac{T_{j}-T_{\mathrm{mean}}}{T_{\mathrm{SD}}},\;$$

where $T_{j}$ represents the regional temperature and $T_{\mathrm{SD}}$ is the sample standard deviation across the five regional values.

The asymmetry index was calculated from the absolute temperature difference between the right and left lateral views, normalized by their average temperature:

(13) $$AI=\frac{|T_{RL}-T_{LL}|}{(T_{RL}+T_{LL})/2}.\;$$

Overall, the participant-level feature set comprised 29 thermal-derived predictors: five original regional temperatures, six regional temperature differences, four temperature ratios, five overall thermal descriptors, three temperature gradients, five standardized regional temperatures, and one asymmetry index. These features were assembled into a single feature vector for each participant and used as predictors in the subsequent machine-learning classification analysis. Periodontal clinical indices were not included as predictors in this thermal-only feature set.

### 2.5. Machine-Learning Dataset and Unit of Observation

The participant was the unit of observation in the machine-learning analysis. For each participant, thermal features derived from the five intraoral views were combined into a single participant-level feature vector independently. During model validation, data partitioning was performed at the participant level so that information from the same participant would not appear in both the training and test sets. All preprocessing and model-tuning procedures were conducted within the training data to minimize information leakage. The machine-learning models were trained using thermal features only; the clinical periodontal parameters were not used as predictor variables for classification. Stratification was used to maintain the representation of healthy controls and advanced periodontitis participants across the folds. No synthetic samples were introduced at any stage of the analysis. All model results were obtained exclusively from the originally measured clinical and thermal data without data augmentation.

### 2.6. Hyperparameter Optimization and Model Evaluation

Eleven machine-learning algorithms were evaluated: Logistic Regression, Support Vector Machine (SVM) with a radial basis function kernel, Random Forest, Extra Trees, AdaBoost, Gradient Boosting, XGBoost, LightGBM, Decision Tree, K-Nearest Neighbors (KNN), and Gaussian Naive Bayes. The candidate hyperparameter configurations considered for these models are summarized in Table 4. The parameters included regularization strength and solver settings for Logistic Regression; kernel and regularization parameters for SVM; tree depth, the number of estimators, and feature-selection settings for tree-based ensemble models; learning rates and other model-specific parameters for boosting algorithms; and neighborhood size and variance-smoothing settings for KNN and Gaussian Naive Bayes, respectively.

Nested five-fold cross-validation was employed to assess the model. In the outer loop, we divided the dataset into five folds, ensuring the class distribution was preserved as closely as possible. For each iteration, four folds were designated for model development and one fold was reserved for evaluation. Within the outer training set, an inner cross-validation procedure was used to evaluate candidate hyperparameter configurations and select the configuration for the corresponding model. The configuration was subsequently adjusted using the full outer training set and subsequently assessed on the separate outer fold. This process was repeated until every fold had served as the outer evaluation set.

The cross-validation process was applied to avoid any information from the evaluation fold that was held out from affecting the fitted transformation. The scaler was first fitted to the training data and then used to transform the corresponding validation or evaluation data. Any preprocessing steps requiring parameter estimation were handled within the appropriate training folds.

The combined predictions from the outer held-out folds were used to determine accuracy, precision, sensitivity, specificity, F1-score, and the area under the receiver operating characteristic curve (ROC-AUC). Confidence intervals were computed to provide a measure of uncertainty for the estimated performance. The algorithms were compared using these measures, with consideration given to the relatively small sample size and the evaluation of multiple models. Although nested cross-validation provides a more structured assessment of model performance when hyperparameters are selected within the inner loop, the resulting estimates remain internal validation results. Independent external validation is required to establish generalizability to other populations and clinical settings.

### 2.7. Statistical Analysis

#### 2.7.1. Descriptive Statistics

All statistical analyses were conducted at the participant level, with each participant treated as an independent observational unit. The distribution of continuous variables was assessed using the Shapiro–Wilk test; normally distributed variables were summarized as mean ± standard deviation, whereas non-normally distributed variables were presented as median and interquartile range. Comparisons between the healthy and advanced periodontitis groups were performed using the independent-samples *t*-test for normally distributed continuous variables and the Mann–Whitney *U* test for non-normally distributed variables. Categorical variables were compared using an appropriate statistical test, depending on the data characteristics.

#### 2.7.2. ROC Curve Analysis and Comparison of Thermal Views

Receiver operating characteristic (ROC) analysis was performed to evaluate the discriminative performance of the five individual intraoral thermal views model. Separate logistic regression models were fitted using the frontal, right lateral, left lateral, maxillary occlusal, and mandibular occlusal temperatures. This model incorporated all five regional temperature variables simultaneously as predictors. Stratified five-fold cross-validation was performed, and out-of-fold predicted probabilities were used to calculate the ROC-AUC for each model. The 95% confidence intervals were estimated using 5000 class-stratified bootstrap resamples of the pooled out-of-fold predictions, with the 2.5th and 97.5th percentiles defining the confidence limits. The AUC values of the individual-view models were compared descriptively to evaluate their discriminative performance across the five intraoral views.

The performance of the 11 machine-learning classifiers was evaluated using nested cross-validation, with the outer cross-validation loop used to estimate generalization performance and the inner loop used for hyperparameter selection. Accuracy, precision, sensitivity, specificity, F1-score, and the area under the receiver operating characteristic curve (ROC–AUC) were used to characterize classification performance. ROC–AUC estimates were reported with 95% confidence intervals. Because cross-validation folds share overlapping training data, fold-level performance scores were not treated as independent observations for definitive statistical comparisons between classifiers. Differences in the observed performance estimates were therefore interpreted descriptively, and no claim of statistical superiority was based solely on the ranking of the classifiers.

## 3. Results

### 3.1. Participant Characteristics and Group Comparisons

Descriptive statistics in Table 5 showed differences in demographic and clinical characteristics between the two groups. Although weight, height, and BMI showed no significant differences between groups, age differed significantly between the groups (p=0.0058). Periodontitis patients also showed significantly higher periodontal indices, including PPD, CAL, and SBI, than healthy controls. Thermal measurements differed across the five intraoral views, indicating potentially relevant thermal patterns associated with periodontal group status. These findings were evaluated further in the subsequent machine-learning analyses.

In contrast, periodontitis patients showed significantly higher periodontal indices, with marked elevations of the PPD, CAL, and SBI values when compared to those of healthy controls. Regarding intraoral thermal measurements, these were also systematically higher for all five intraoral views in the periodontitis group, reflecting stronger inflammatory heat patterns. These between-group differences indicate that the measured thermal features may contain discriminatory information in the subsequent ML classification.

There was a statistically significant difference in age between the groups, with a (p=0.0058), as shown in Table 5. The periodontal indicators (PPD, CAL, and SBI) differed significantly between the groups (p<0.001). All five intraoral thermal views also showed statistically significant between-group differences. However, the observed age difference may have contributed to the thermal differences or classification performance, and its potential confounding effect requires further evaluation.

#### Distribution Patterns of Clinical and Thermal Views

**Figure 4:**
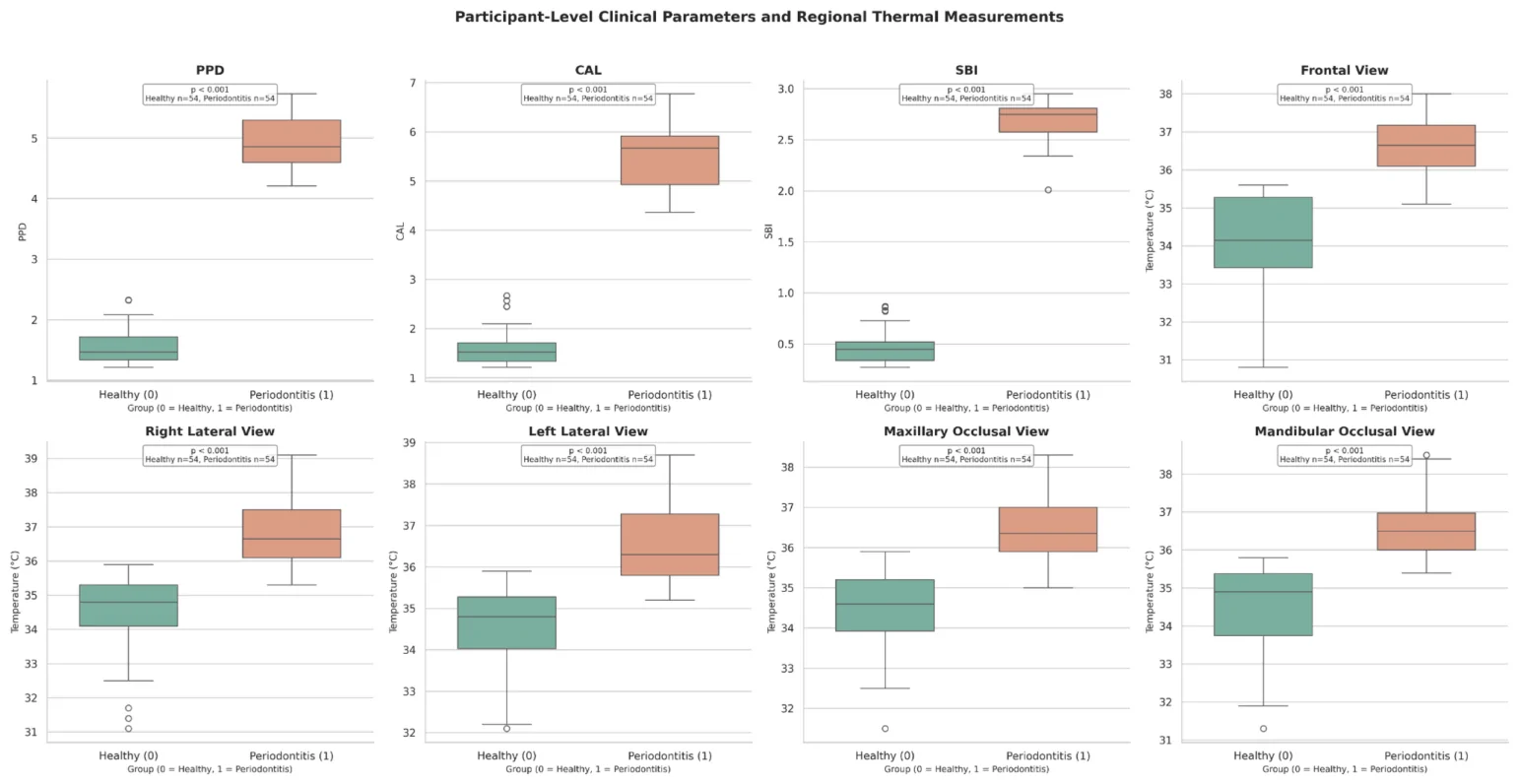
Distribution of thermographic characteristics and periodontal clinical indicators in healthy controls and patients with advanced periodontitis.

Figure 4 shows the distribution of major periodontal and thermal characteristics in healthy individuals compared with participants with advanced periodontitis. The first row illustrates the clinical indices, namely, PPD, CAL, and SBI. Each of them is shown to have a clearly elevated and tightly clustered distribution in the periodontitis group, having no overlap with the healthy group and thereby reflecting the expected pathological increase in probing depth, attachment loss, and gingival bleeding. The following figures show the temperature-based biomarkers acquired from the five intraoral locations of interest. The observed thermal measurements were generally higher in the advanced-periodontitis group, indicating localized inflammatory activity and enhanced mucosal heat signatures.

In all thermal ROIs, the density curves for periodontitis are shifted upwards with broader dispersion, while healthy individuals show narrower and lower temperature ranges. Collectively, the box plots visually confirm the statistical results by demonstrating clear between-group separation in the observed distributions in both clinical and thermal domains. This would further suggest the screened utility of thermal imaging as a supplementary indicator for advanced periodontal disease.

### 3.2. ROC–AUC Performance of Clinical Thermal Features

ROC analysis was performed to evaluate the ability of individual intraoral thermal views and the final machine-learning model to distinguish healthy participants from those with advanced periodontitis. The ROC curve illustrates the relationship between the true positive rate (sensitivity) and the false positive rate (1 − specificity) across different classification thresholds. The area under the ROC curve (AUC) provides an overall measure of discrimination, with values closer to 1.0 indicating better separation between the two groups, whereas an AUC of 0.5 represents performance equivalent to random classification.

**Figure 5:**
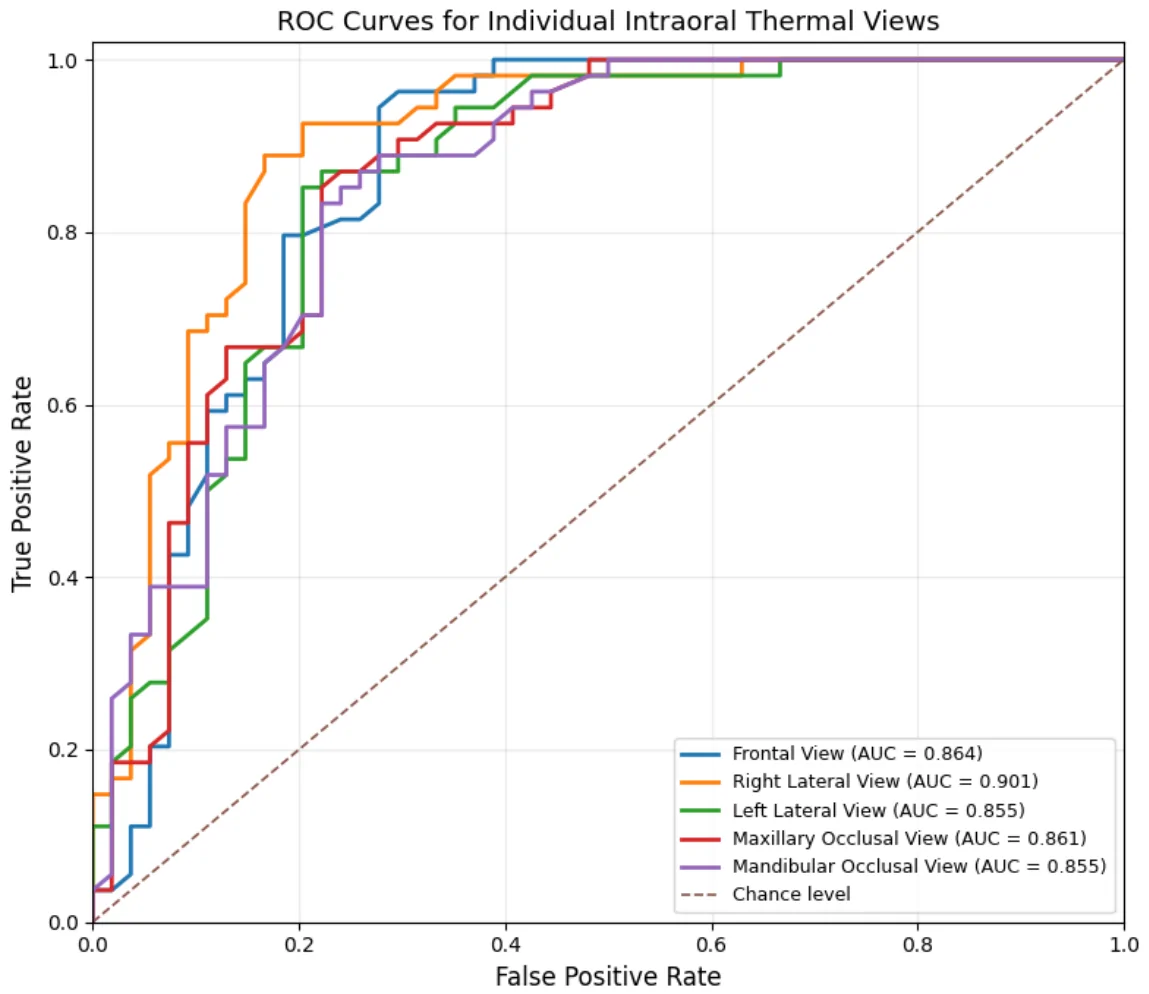
ROC curves for individual intraoral thermal views with their respective AUC values.

As shown in Figure 5, the five individual intraoral thermal views demonstrated good discrimination between healthy controls and participants with advanced periodontitis. The right lateral view achieved the highest individual-view ROC-AUC (0.901; 95% CI: 0.832–0.956), followed by the frontal view (0.864; 95% CI: 0.783–0.930), maxillary occlusal view (0.861; 95% CI: 0.782–0.927), left lateral view (0.855; 95% CI: 0.775–0.922), and mandibular occlusal view (0.855; 95% CI: 0.779–0.921). The combined five-view model, incorporating all five regional temperatures simultaneously, achieved an ROC-AUC of 0.900 (95% CI: 0.827–0.958). Comparable to the highest individual-view estimate obtained for the right lateral view (AUC = 0.901; 95% CI: 0.832–0.956). The combined model had higher AUC estimates than the other four individual views; however, these differences were interpreted descriptively and do not establish superior discrimination. These comparisons should be interpreted cautiously because the bootstrap analysis was conditional on the pooled out-of-fold predictions and the nominal *p*-values do not remain statistically significant after adjustment for multiple comparisons.

PPD, CAL, and SBI were excluded from the ROC–AUC comparison in Figure 5 because these clinical parameters were closely associated with the criteria used to classify participants into the study groups. Their discrimination could therefore reflect the group-definition process rather than independent screening performance.

These findings should be interpreted in the context of the study design. The analysis involved a comparison between healthy participants and participants with advanced periodontitis, rather than the full clinical spectrum of periodontal disease. Accordingly, the ROC results represent exploratory discrimination within the study sample and should not be interpreted as evidence of validated screening performance in routine clinical practice. Evaluation in larger, independent cohorts that include different stages of periodontal disease is required to establish the generalizability and clinical utility of these findings.

### 3.3. Correlation Heatmap

**Figure 6:**
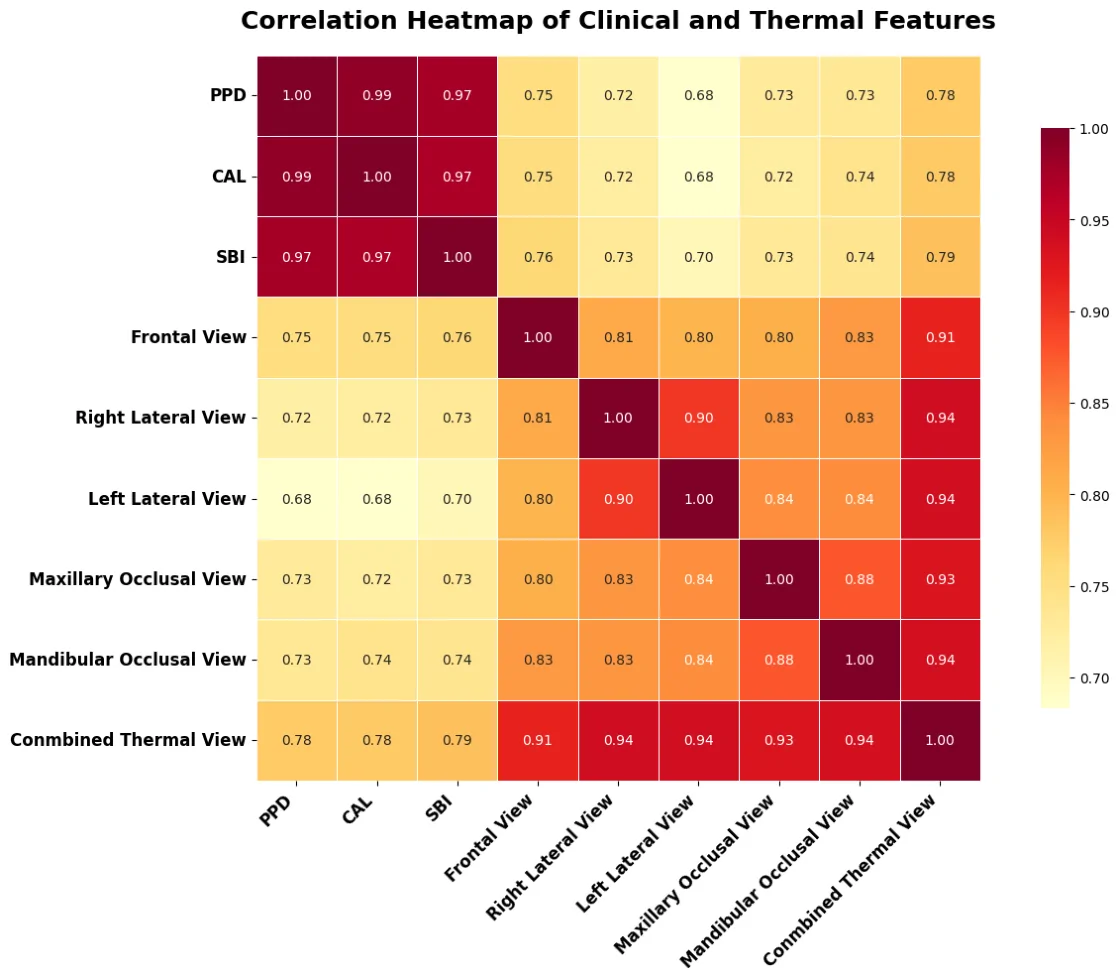
Spearman correlation heatmap of clinical periodontal indices and intraoral thermal measurements.

Figure 6 Spearman correlation analysis revealed strong pairwise correlations among the clinical periodontal variables PPD, CAL, and SBI ($r_{s}=0.97$–1.00) and between the combined thermal measure and the five individual intraoral views ($r_{s}=0.91$–0.94). Strong correlations were also observed between the right and left lateral views ($r_{s}=0.90$) and between the maxillary and mandibular occlusal views ($r_{s}=0.88$). Group-specific analyses further characterized the clinical–thermal relationships within healthy controls and participants with advanced periodontitis; however, none of the 15 clinical–thermal associations in either group remained statistically significant after Holm correction for multiple comparisons. These findings highlight the strong interrelationships among clinical measures and the consistency of thermal measurements across intraoral views, while suggesting that pooled clinical–thermal correlations may partly reflect differences between the study groups.

### 3.4. Performance of Machine Learning Models

#### 3.4.1. Performance Metrics

The classification performance was evaluated using standard statistical measures defined as follows:

(14) $$\mathrm{Accuracy}=\frac{TP+TN}{TP+TN+FP+FN}$$

(15) $$\mathrm{Sensitivity}\;\left(\mathrm{Recall}\right)=\frac{TP}{TP+FN}$$

(16) $$\mathrm{Specificity}=\frac{TN}{TN+FP}$$

(17) $$\mathrm{Precision}=\frac{TP}{TP+FP}$$

(18) $$F1\text{-}\mathrm{score}=2\times \frac{\mathrm{Precision}\times \mathrm{Recall}}{\mathrm{Precision}+\mathrm{Recall}}$$

#### 3.4.2. Classification Performance Using Thermal-Derived Features

Eleven machine-learning classifiers were evaluated to examine the ability of 29 thermal-derived features to distinguish healthy participants from those with advanced periodontitis. The feature set comprised temperature measurements from five intraoral views, inter-view temperature differences and ratios, thermal distribution descriptors, temperature gradients, standardized regional temperatures, and an asymmetry index. Because the five views were obtained from the same participant, the participant was treated as the unit of analysis.

Nested cross-validation was used to evaluate model performance, with the outer loop used for performance estimation and the inner loop used for hyperparameter optimization. The results are presented in Table 6, in descending order of classification accuracy. AdaBoost achieved the highest observed accuracy of 98.15%, with precision, sensitivity, specificity, and F1-score also reported as 98.15% from Equations (14)–(18). Random Forest achieved an accuracy of 97.22%, with a sensitivity of 96.30% and specificity of 98.15%. XGBoost achieved an accuracy of 96.30% and sensitivity of 98.15%, with a specificity of 94.44%.

Gradient Boosting and LightGBM each achieved an accuracy of 95.37%, followed by Logistic Regression at 94.44%. Extra Trees and Decision Tree each achieved 93.52%, while the radial basis function support vector machine (SVM), Gaussian Naive Bayes, and K-nearest neighbours (KNN) achieved accuracies of 91.67%, 90.74%, and 87.04%, respectively. These results indicate that the observed classification performance varied across the evaluated learning algorithms. Table 6, the reported ROC-AUC estimates ranged from 0.941 for Decision Tree to 0.998 for AdaBoost. Overall, the findings suggest that thermal-derived features may support exploratory discrimination between healthy participants and participants with advanced periodontitis.

## 4. Discussion

This study investigated the potential of intraoral thermal imaging and machine-learning methods to distinguish healthy participants from those with advanced periodontitis. The findings showed higher temperatures across all five intraoral views in the advanced-periodontitis group compared with the healthy group. The results suggest that regional thermal measurements may provide useful information for exploring differences in periodontal health status.

The present findings can be considered alongside previous investigations of thermal imaging in periodontal and related dental conditions, as summarized in Table 1. Aboushady et al. [7] investigated thermal differences associated with periapical inflammatory lesions, providing related evidence that dental inflammatory conditions can be accompanied by measurable thermal variation. Elboray et al. [16] examined the discriminatory potential of thermal measurements in relation to periodontal inflammation and attachment-loss sites. In a related direction, Çankaya et al. [18] explored machine-learning classification of gingival inflammation using thermal images. Morales-Cervantes et al. [19] combined facial thermal measurements with clinical variables for periodontal classification, illustrating the potential value of integrating thermal information with other clinical measurements. Although these studies differ in imaging location, clinical targets, feature construction, and analytical methods, collectively they provide a rationale for investigating thermography as a complementary source of information in periodontal assessment.

The quantitative findings of the present study further support the potential relevance of intraoral thermal measurements in distinguishing healthy participants from those with advanced periodontitis. Mean temperatures were significantly higher in the advanced-periodontitis group across all five intraoral views, with between-group differences ranging from approximately 1.98 °C to 2.40 °C. The consistency of these differences across the frontal, lateral, maxillary, and mandibular views suggests that the observed thermal variation was not limited to a single imaging region.

The individual-view ROC analysis provided further insight into the discriminatory potential of the thermal measurements. The right lateral view achieved the highest AUC (0.901; 95% CI: 0.832–0.956), while the frontal, left lateral, maxillary occlusal, and mandibular occlusal views also demonstrated discriminatory ability. Yet the right lateral view showed the highest observed AUC, the results do not establish statistically significant superiority over the other views. These findings suggest that thermal patterns from different intraoral regions may provide complementary information, although further investigation is required to determine the most reproducible and informative imaging locations.

The machine-learning analysis also demonstrated promising classification performance. AdaBoost achieved the highest observed accuracy of 98.15%, followed by Random Forest at 97.22% and XGBoost at 96.30%. These results indicate that the extracted thermal features contained information useful for distinguishing the two study groups under the evaluated validation procedure. Nevertheless, high internal classification performance does not necessarily translate into equivalent performance in clinical practice. The findings should therefore be considered exploratory until the models are evaluated using independent datasets representing a broader range of periodontal conditions and patient characteristics. The correlation analysis provided an additional perspective on the relationship between thermal measurements and periodontal clinical parameters. The strong correlations were observed among the clinical measures and among thermal measurements from different intraoral views.

Several methodological considerations should be acknowledged when interpreting these results. The study compared healthy participants with individuals who had Stage III or Stage IV periodontitis, without including participants with Stage I or Stage II disease. This restricted disease spectrum may result in more favourable discrimination than would be observed in a general clinical population. The groups also differed significantly in age, which may have influenced the observed thermal patterns. In addition, periodontal measurements were summarized at the participant level, whereas thermal features were extracted from separate intraoral views, preventing direct anatomical matching between individual clinical measurements and thermal regions. Manual ROI selection and the absence of quantitative intra- and inter-observer reliability assessment may also affect reproducibility. Finally, blackbody calibration and independent emissivity validation were not performed, which should be considered when interpreting absolute temperature measurements.

Overall, these findings highlight the potential of intraoral thermal imaging combined with machine-learning analysis as a complementary, non-contact screening tool alongside conventional clinical periodontal assessment. Further research involving a broader spectrum of periodontal conditions, standardized anatomical region-selection procedures, formal reproducibility assessments, and independent multicentre validation is warranted to establish the generalizability and clinical utility of this approach.

## 5. Conclusions

This study demonstrated significantly higher intraoral temperatures across all five views in individuals with advanced periodontitis compared with healthy controls. Individual thermal views and machine-learning classifiers demonstrated promising discriminatory performance, with AdaBoost achieving the highest observed classification accuracy. Overall, these findings highlight the potential of quantitative intraoral thermal imaging combined with machine-learning analysis as a complementary tool for conventional periodontal assessment and support further research toward its clinical application.

## 6. Patents

This study is associated with Indian Patent Application No. 202541082282 A, entitled “A Non-Invasive System for Early Detection and Classification of Periodontal Abnormalities,” filed with the Indian Patent Office on 29 August 2025 and published on 26 September 2025. SRM University is listed as the applicant, and Ratna Bhaskar Pallipamula, Anantha Sunitha Karnam, Ravindranath Dhulipalla, and Kishore Kumar Katuri are listed as inventors. The patent describes a broader non-invasive system involving intraoral thermal imaging, image preprocessing, segmentation, feature extraction, artificial intelligence-based analysis, and classification. The present study evaluates the specified thermal-derived features and machine-learning classifiers using the collected participant dataset; it does not constitute experimental validation of every component or the full scope of the patented invention. The patent application is disclosed to ensure transparency regarding the intellectual property associated with this research. 

## Figures and Tables

**Table 2:** Clinical Criteria for Group Classification and Thermal Imaging Assessment.

Clinical Parameter	Advanced Periodontitis	Healthy Controls	Thermal Imaging Assessment
**Probing Pocket Depth (PPD) [20]**	≥6 mm	≤3 mm	Quantitative thermal features extracted from five intraoral views for both groups.
**Clinical Attachment Loss (CAL) [20]**	≥5 mm	≤3 mm	
**Sulcus Bleeding Index (SBI) [21]**	2–5	0–1	

**Table 3:** Thermal Images of Temperature Variations in Periodontitis and Healthy Subjects.

S. No	Oral Region	Periodontitis Subjects	Healthy Subjects
1	Frontal View	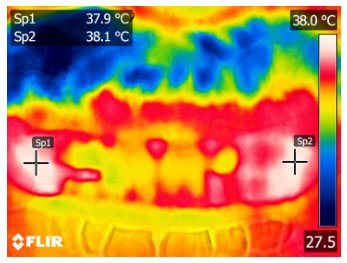	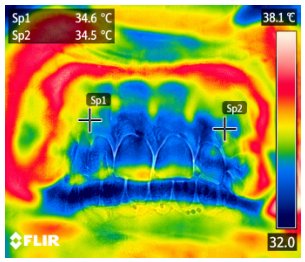
2	Right Lateral View	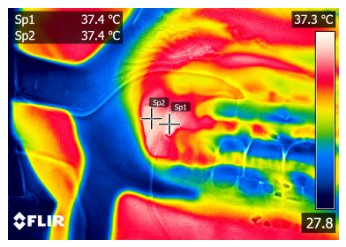	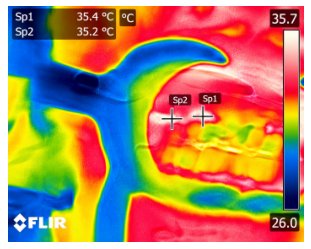
3	Left Lateral View	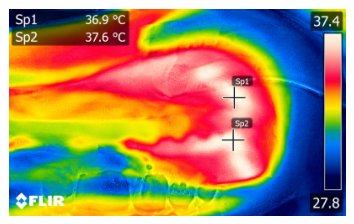	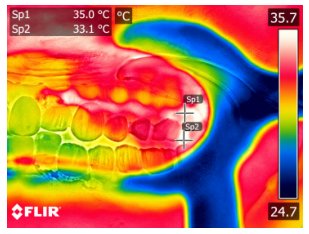
4	Maxillary Occlusal View	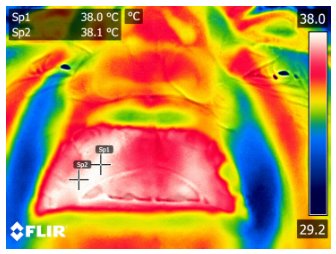	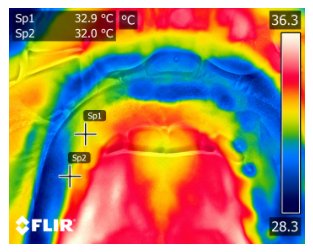
5	Mandibular Occlusal View	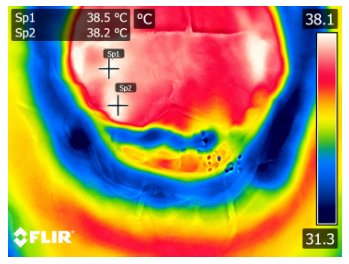	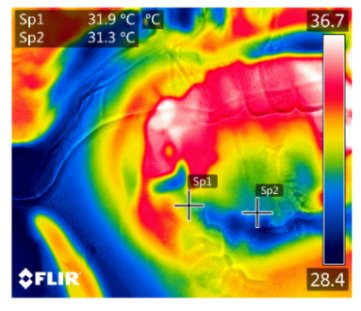

**Table 4:** Hyperparameter configurations used for the eleven machine-learning models.

Model	Parameter(s)	Tested Values	Selected Value
Logistic Regression	Regularization strength (*C*)	{0.1,1,10}	C=10; penalty = L2; solver = LBFGS; max_iter = 2000
SVM (RBF)	*C*, kernel, γ, probability	C=10; kernel = RBF; γ=scale; probability = True	C=10; kernel = RBF; γ=scale; probability = True
Random Forest	max_depth, n_estimators	max_depth: {None,5,10,20}; n_estimators: {100,200,400,600}	max_depth = 20; n_estimators = 200; max_features = sqrt
Extra Trees	max_depth, n_estimators	max_depth: {5,10,20}; n_estimators = 200	max_depth = 20; n_estimators = 200; random_state = 42
AdaBoost	learning_rate, n_estimators	learning_rate: {0.01,0.1,0.8,1}; n_estimators: {50,100,200,300}	learning_rate = 0.8; n_estimators = 200
Gradient Boosting	learning_rate, n_estimators, max_depth	n_estimators = 200; learning_rate = 0.01; max_depth = 3	learning_rate = 0.01; n_estimators = 200; max_depth = 3
XGBoost	max_depth, n_estimators, subsample	max_depth: {3,4,5,6}; n_estimators: {100,200,250,300}; subsample = 0.8	max_depth = 4; n_estimators = 200; subsample = 0.8; eval_metric = logloss
LightGBM	learning_rate, n_estimators, num_leaves	learning_rate: {0.01,0.1,0.5,1}; num_leaves: {15,31,63}; n_estimators = 200	learning_rate = 0.5; n_estimators = 200; num_leaves = 31
Decision Tree	criterion, max_depth	criterion = log_loss; max_depth = 10	criterion = log_loss; max_depth = 10
K-Nearest Neighbors	n_neighbors, weights	n_neighbors = 5; weights = distance	n_neighbors = 5; weights = distance
Gaussian Naive Bayes	var_smoothing	$\left\{10^{-8}\right\}$	var_smoothing = $10^{-8}$

**Table 5:** Comparison of Demographic, Clinical and Intraoral Thermal Parameters Between Advanced Periodontitis and Healthy Controls.

Parameter	Advanced Periodontitis			Healthy Controls			*p*-Value
	**Mean ± SD**	**IQR**	**Range (Min–Max)**	**Mean ± SD**	**IQR**	**Range (Min–Max)**	
**Demographic and anthropometric characteristics**							
Age (years)	43.81±6.89	12.0	39–66	40.28±6.00	8.0	35–58	0.00577
Weight (kg)	66.67±12.87	15.25	45–115	69.48±15.13	15.75	35–110	0.30464
Height (ft)	5.29±0.25	0.43	4.90–5.90	5.37±0.30	0.5	4.80–5.90	0.12769
BMI (kg/m^2^)	25.59±4.36	4.26	15.05–38.05	25.93±5.63	5.21	15.17–43.80	0.72983
**Periodontal clinical parameters**							
PPD (mm)	5.50±0.56	0.74	4.36–6.77	1.62±0.40	0.44	1.21–2.67	<0.001
CAL (mm)	4.90±0.42	0.99	4.21–5.73	1.56±0.31	0.43	1.20–2.33	<0.001
SBI	2.68±0.20	0.25	2.01–2.95	0.46±0.18	0.19	0.27–0.87	<0.001
**Thermal View Features**							
Frontal View (°C)	36.64±0.66	1.08	35.10–38.0	34.24±1.57	1.98	30.80–37.90	<0.001
Right Lateral View (°C)	36.82±0.98	1.43	34.60–39.10	34.50±1.15	1.20	31.10–37.10	<0.001
Left Lateral View (°C)	36.52±1.04	1.60	34.60–38.70	34.54±1.09	1.43	32.10–36.50	<0.001
Maxillary Occlusal View (°C)	36.56±0.88	1.13	35.0–38.30	34.53±1.02	1.43	31.50–36.10	<0.001
Mandibular Occlusal View (°C)	36.58±0.79	1.03	35.10–38.50	34.54±1.14	1.83	31.30–36.90	<0.001

**Table 6:** Internal validation performance of machine-learning models using nested cross-validation with 29 thermal-derived features.

Model	Accuracy	Precision	Sensitivity	Specificity	F1-Score	ROC-AUC (95% CI)
AdaBoost	98.15	98.15	98.15	98.15	98.15	0.998 (0.992–1.000)
Random Forest	97.22	98.11	96.30	98.15	97.20	0.996 (0.989–1.000)
XGBoost	96.30	94.64	98.15	94.44	96.36	0.996 (0.987–1.000)
Gradient Boosting	95.37	92.98	98.15	92.59	95.50	0.997 (0.990–1.000)
LightGBM	95.37	94.55	96.30	94.44	95.41	0.996 (0.988–1.000)
Logistic Regression	94.44	96.15	92.59	96.30	94.34	0.989 (0.970–1.000)
Extra Trees	93.52	94.34	92.59	94.44	93.46	0.993 (0.981–1.000)
Decision Tree	93.52	94.34	92.59	94.44	93.46	0.941 (0.893–0.980)
SVM (RBF)	91.67	92.45	90.74	92.59	91.59	0.977 (0.951–0.995)
Gaussian NB	90.74	86.67	96.30	85.19	91.23	0.973 (0.945–0.992)
KNN (k=5)	87.04	85.71	88.89	85.19	87.27	0.961 (0.928–0.985)

## Data Availability

The data generated and analyzed during this study are not publicly available because they contain participant-related clinical information and are subject to ethical and privacy restrictions. De-identified data may be made available by the corresponding author upon reasonable request, subject to approval by the relevant institutional ethics committee and applicable privacy requirements.
